# NH_2_-MIL-53(Al) with Hierarchical Micro–Mesoporosity for Highly Efficient Capture of Direct Scarlet Anionic Dye: Insights into Structure–Property Correlations

**DOI:** 10.3390/nano16181135

**Published:** 2026-09-10

**Authors:** Ruiming Zhao, Lingling Li

**Affiliations:** 1 Center of Analysis and Testing, Guangdong University of Petrochemical Technology, Maoming 525000, China; rmzhao@gdupt.edu.cn; 2 School of Chemical Engineering, Guangdong University of Petrochemical Technology, Maoming 525000, China

**Keywords:** NH_2_-MIL-53(Al), hierarchical porous MOF, anionic dye, electrostatic adsorption, water remediation

## Abstract

Fabricating nanoscale metal–organic frameworks (MOFs) with hierarchical pores is an effective strategy to engineer high-performance adsorbents. Herein, hierarchically porous NH_2_-MIL-53(Al) nanorods were synthesized through a straightforward one-pot solvothermal approach, featuring inherently interconnected micro-/mesoporous with an average pore width of 19.51 nm and abundant amino-functionalized active sites. Toward the anionic Direct Scarlet dye, the material delivers a Langmuir maximum adsorption capacity of 694.99 mg·g^−1^, with its adsorption kinetics and isotherms well described by the pseudo-second-order kinetics and Langmuir isotherm model. Combined spectroscopic, thermodynamic, and diffusion analyses suggest monolayer electrostatic chemisorption between protonated –NH_3_^+^ and dye sulfonate groups. The material exhibits favorable reusability, retaining over 85% of its original dye removal efficiency after five adsorption–desorption cycles, and PXRD and SEM results verify well-preserved morphology and crystalline lattice. This work clarifies the intrinsic correlation between structure and adsorption performance of NH_2_-MIL-53(Al), offering a facile nanoscale pore-tuning strategy for the design and fabrication of high-performance MOF adsorbents.

## 1. Introduction

Textile and cosmetic industries continuously discharge massive volumes of wastewater containing hazardous anionic azo dyes [1,2]. Untreated residual dyes are toxic and potentially carcinogenic, posing substantial risks to ecological systems and human health [3,4]. To address dye contamination, diverse water remediation techniques have been developed, such as biological degradation, membrane separation, advanced oxidation, and electrochemical treatment. Among these alternatives, adsorption is regarded as one of the most feasible and practical strategies, owing to its low operational cost, simple implementation, and negligible secondary pollution [5,6]. Traditional adsorbents such as activated carbon and metal oxide microparticles are confined by single microporous frameworks, which hinder the inward diffusion of large dye molecules and limit saturated adsorption capacity [7].

Metal–organic frameworks (MOFs) have garnered substantial attention as promising candidates for dye removal, owing to their exceptional specific surface area, high chemical stability, tunable functional groups, and abundant accessible active sites [8,9,10,11,12]. As a classic aluminum-based MOF constructed from Al^3+^ clusters and terephthalate ligands, MIL-53(Al) has been widely adopted in environmental purification research due to its outstanding water resistance and superior thermal and chemical stability [13,14]. Pristine MIL-53 (Al) can capture anionic azo dyes, yet it suffers from micropore-induced diffusion limitations [15]. Amino-functionalized MOFs are promising for azo-dye removal. Nevertheless, conventional microporous NH_2_-MIL-53(Al) still encounters this bottleneck; bulky azo dyes hardly access inner pores, and adsorption mainly occurs on crystal outer surfaces, leading to low pore utilization [16]. Other amino-modified MOFs such as NH_2_-MIL-101(Al) achieve high capacity but exhibit poor long-term aqueous stability [17]. The intrinsic microporous structure of MOFs inevitably impedes mass transfer and restricts the diffusion of relatively large organic dye molecules into the internal active sites [7,18]. To address this drawback, the construction of hierarchically porous architectures by introducing mesopores or macropores into the MOF framework has become an effective and widely used strategy [19,20].

Defective NH_2_-UiO-66 nanocrystals were synthesized for dye capture in our previously published research [21]. Nevertheless, discrete defect-derived cavities fail to construct interconnected mass-transport pathways, restricting adsorption kinetics and cycling stability. Different from the defect-induced mesopore route, hierarchically porous NH_2_-MIL-53(Al) was synthesized via a one-pot solvothermal approach in this study, and its performance for the removal of Direct Scarlet dye from aqueous solutions was systematically evaluated. The synthesized material was comprehensively characterized to confirm its crystal structure, porous features, and thermal stability, revealing the formation of a well-defined hierarchically porous architecture. Adsorption performance was systematically explored by adjusting key experimental variables, including adsorbent dosage, contact time, initial dye concentration, and solution temperature. Detailed analyses of adsorption kinetics, isotherms, and thermodynamics verified its excellent removal capability toward Direct Scarlet. Multiple adsorption–desorption cycling tests were further implemented to evaluate the reusability and structural stability. Combined with spectroscopic characterization, a reasonable adsorption mechanism was proposed, which reveals the synergistic contribution between hierarchical nanochannel structure and surface amino functional groups. This study provides a practical reference for the rational design and fabrication of stable hierarchically porous MOF adsorbents for efficient remediation of dye-contaminated wastewater.

## 2. Materials and Methods

### 2.1. Chemicals and Raw Materials

Nonahydrated aluminum nitrate (Al(NO_3_)_3_·9H_2_O, 98%), terephthalic acid (H_2_BDC) and 2-aminoterephthalic acid (NH_2_-BDC, ≥98% purity), N,N-dimethylformamide (DMF), anhydrous methanol, and Direct Scarlet anionic dye were supplied by J&K Scientific (Shanghai, China)and Sinopharm Chemical Reagent Co., Ltd. (Shanghai, China). All raw chemicals were used directly without further purification. Ultra-pure water with 18.2 MΩ·cm resistivity was produced via a Merck Millipore water purification instrument.

### 2.2. Synthesis of Hierarchically Porous NH_2_-MIL-53(Al) Nanocrystals

A facile one-pot solvothermal strategy was adopted to fabricate hierarchically NH_2_-MIL-53(Al), without introducing any modulator or artificial linker defects [22]. Equimolar aluminum nitrate nonahydrate and NH_2_-BDC powders were dispersed in 40 mL of DMF, followed by 20 min of ultrasonication to eliminate agglomeration and form a homogeneous precursor suspension. The uniform mixture was transferred into a 100 mL PTFE-lined stainless steel autoclave and subjected to crystallization at 220 °C for 24 h.

After natural cooling to room temperature, the crystalline precipitates were collected via centrifugation at 8000 rpm for 10 min. The crude product was repeatedly rinsed with DMF and anhydrous methanol to remove surface-adsorbed metal ions and uncoordinated free ligands. The purified powder was vacuum-dried at 100 °C for 24 h to fully eliminate residual solvent trapped within the nanochannels. The final rod-like nanocrystals were sealed and stored at ambient temperature for subsequent characterization and adsorption experiments.

### 2.3. Material Characterizations

The crystal structure of the prepared NH_2_-MIL-53(Al) was characterized via powder X-ray diffraction (XRD, Rigaku-Ultima IV, Rigaku Corporation, Tokyo, Japan) with Cu-Kα radiation. The scanning range was set from 5° to 60° at a rate of 8° min^−1^, and the diffraction patterns of fresh and dye-adsorbed samples were compared to evaluate lattice structural integrity. Surface morphology and microscopic dispersion were observed using field-emission scanning electron microscopy (FE-SEM, Regulus 8220, Hitachi High-Tech Corporation, Tokyo, Japan), where all powder specimens were pre-coated with a thin gold film to eliminate charge accumulation during testing. Fourier transform infrared spectroscopy (FT-IR, Nicolet iS50, Thermo Fisher Scientific, Waltham, MA, USA) was performed via the KBr pellet method within 4000–400 cm^−1^ to identify surface functional groups and coordination bond variations before and after adsorption. Nitrogen adsorption–desorption measurements were carried out at 77 K on a Micromeritics ASAP 2020 instrument(Micromeritics Instrument Corporation, Norcross, GA, USA). All samples were degassed at 150 °C overnight prior to testing. The obtained isotherm data were further analyzed to determine the BET specific surface area, pore volume, and average nanochannel size; detailed calculation procedures for micropore/mesopore size distribution and pore-parameter evaluation are provided in the Supporting Information. Thermal stability was examined on a simultaneous thermal analyzer(STA 449F3, NETZSCH-Gerätebau GmbH, Selb, Bavaria, Germany). The samples were heated from 25 °C to 800 °C at a heating rate of 10 K min^−1^ under continuous air flow. Thermogravimetric curves were analyzed to distinguish solvent desorption and ligand pyrolysis stages, further verifying the structural robustness of the intrinsic Al-O framework.

### 2.4. Batch Adsorption Experiments

All adsorption experiments were conducted under neutral aqueous conditions without additional pH adjustment. To systematically explore the dye removal performance of hierarchically porous NH_2_-MIL-53(Al), multiple experimental variables were optimized, including adsorbent dosage (2.5–40 mg), contact time (10–400 min), initial Direct Scarlet concentration (100–800 mg·L^−1^), and reaction temperature (298 K, 308 K, 318 K). All adsorption experiments were performed in triplicate independent runs, and the reported experimental data are presented as the mean values. After reaching adsorption equilibrium, solid–liquid separation was achieved by centrifugation, and the residual concentration of dye in the supernatant was determined via UV–vis spectrophotometry.

The equilibrium adsorption capacity and dye removal efficiency were calculated using standard formulas provided in the Supporting Information. Time-dependent adsorption data were fitted with pseudo-first-order, pseudo-second-order, and Weber–Morris intra-particle diffusion kinetic models. Equilibrium data obtained at different temperatures were further analyzed by Langmuir, Freundlich, and Temkin isotherm models. Meanwhile, the distribution coefficient derived from equilibrium results was applied to calculate thermodynamic parameters (ΔG, ΔH, ΔS), aiming to evaluate the spontaneity and thermal characteristics of the adsorption process.

### 2.5. Adsorbent Regeneration and Cyclic Stability Tests

To evaluate the practical reusability and structural durability of hierarchically porous NH_2_-MIL-53(Al), continuous adsorption–desorption cycling experiments were carried out. After each saturated adsorption process, the solid adsorbent was separated by centrifugation and repeatedly rinsed with a 70% ethanol aqueous solution to effectively elute Direct Scarlet molecules trapped within the multiscale nanochannels [23]. The treated powder was dried at 100 °C to completely remove residual ethanol and then reused for the next adsorption cycle. A total of five consecutive cyclic tests were performed. After each regeneration round, the residual dye concentration in the supernatant was measured via UV–vis spectrophotometry, and the corresponding dye removal efficiency was recorded. The cycling results further evaluated the short-term laboratory-scale reusability of the lattice-intact crystalline nanoadsorbent.

## 3. Results and Discussion

### 3.1. Structural, Morphological, and Porous Characterizations

**Figure 1:**
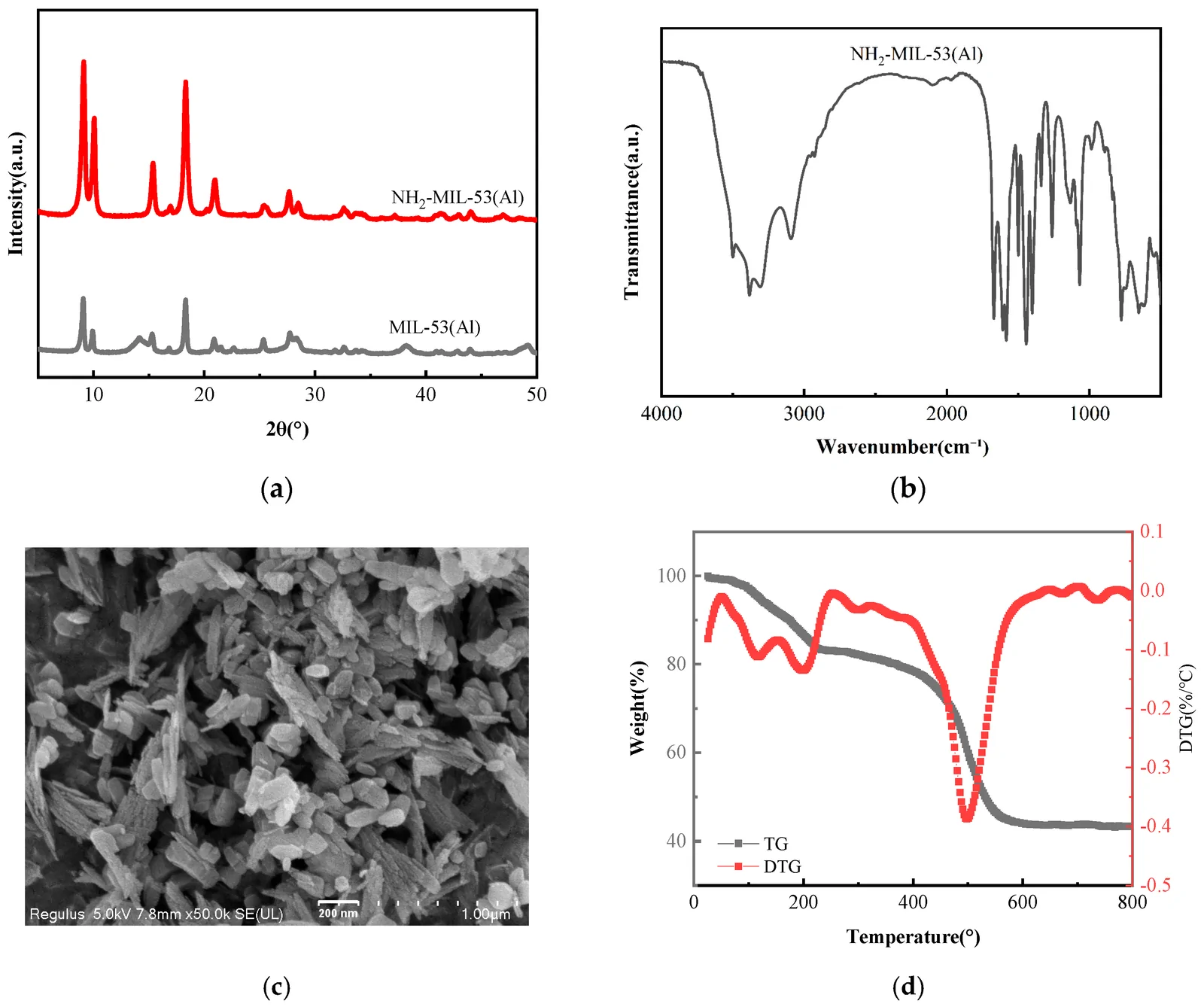
(**a**) XRD patterns, (**b**) FT-IR spectra, (**c**) SEM images, and (**d**) TGA and DTG curves of NH_2_-MIL-53(Al).

The crystallinity and phase purity of the as-synthesized NH_2_-MIL-53(Al) were verified by powder XRD measurement, as displayed in Figure 1a. The distinct diffraction peaks observed at 2θ = 9.2°, 12.5°, 15.1°, 18.2°, 21.1°, 25.3°, 28.2°, and 32.6° are well consistent with the standard orthorhombic crystal data (CCDC No. 220477) and previously reported NH_2_-MIL-53(Al) patterns [22,24]. Specifically, the characteristic peak at 9.2° corresponds to the (110) crystal plane, while the peak at 18.2° is attributed to the overlapping reflection of (211) and (220) crystal planes. No miscellaneous diffraction signals were detected, confirming the successful fabrication of high-purity and well-crystallized NH_2_-MIL-53(Al) via the one-pot solvothermal route without defect modulation.

FTIR spectroscopy was further employed to identify surface functional groups of the material, as shown in Figure 1b. The absorption bands located at 1609 cm^−1^ and 1403 cm^−1^ are assigned to the asymmetric and symmetric stretching vibrations of carboxylate groups, confirming the effective coordination between NH_2_-BDC organic linkers and Al^3+^ metal clusters [25]. The characteristic peaks at 1583 cm^−1^ and 1440 cm^−1^ originate from the C=C stretching vibration of benzene rings, and the absorption at 778 cm^−1^ corresponds to the benzene ring C–H bending vibration. The broad absorption band centered at 3390 cm^−1^ is attributed to N–H stretching of surface amino groups, verifying the successful introduction of amino functional sites on the nanochannel walls. In addition, the weak band at 1700 cm^−1^ derives from residual unreacted NH_2_-BDC ligands, and the peak at 994 cm^−1^ is associated with surface hydroxyl groups [26]. These characteristic spectral features are consistent with the typical structural properties of amino-functionalized MIL-53(Al) materials, further validating the successful synthesis of the target adsorbent.

Microscopic morphology and particle dispersion status of the prepared samples were observed using FE-SEM (Figure 1c). The obtained NH_2_-MIL-53(Al) presents uniform prismatic nanorod features with complete geometric structure and narrow particle size distribution, which is in line with the typical morphological characteristics of MIL-53 series materials [13,14,22]. Benefiting from the solvent-only synthesis system without any defect regulators, the prepared nanorods display good dispersion integrity. No serious particle agglomeration, structural distortion, or surface collapse was observed in the microscopic field of view. Such uniform and stable micro-morphology reflects excellent structural homogeneity and crystal quality, which provides a stable structural basis for repeated adsorption applications.

TG analysis was conducted to evaluate the thermal stability of the crystalline framework, as plotted in Figure 1d. The thermal decomposition process of the material can be divided into three obvious stages, with a total mass loss of 54% within 25–800 °C. The minor mass loss of approximately 3% below 100 °C was caused by the desorption of physically adsorbed water on the material surface. The mass loss of 14% occurring between 100 °C and 250 °C was related to the removal of residual DMF solvent trapped inside the framework gaps. When exceeding 410 °C, a sharp mass loss of 40% emerged, which corresponded to the intense thermal decomposition of amino-containing organic ligands, and the final residual substance was amorphous alumina [13,22]. Notably, the integral framework structure remained intact below 400 °C, revealing outstanding thermal stability. Such excellent high-temperature structural robustness enables the material to maintain stable performance under conventional wastewater treatment conditions.

**Figure 2:**
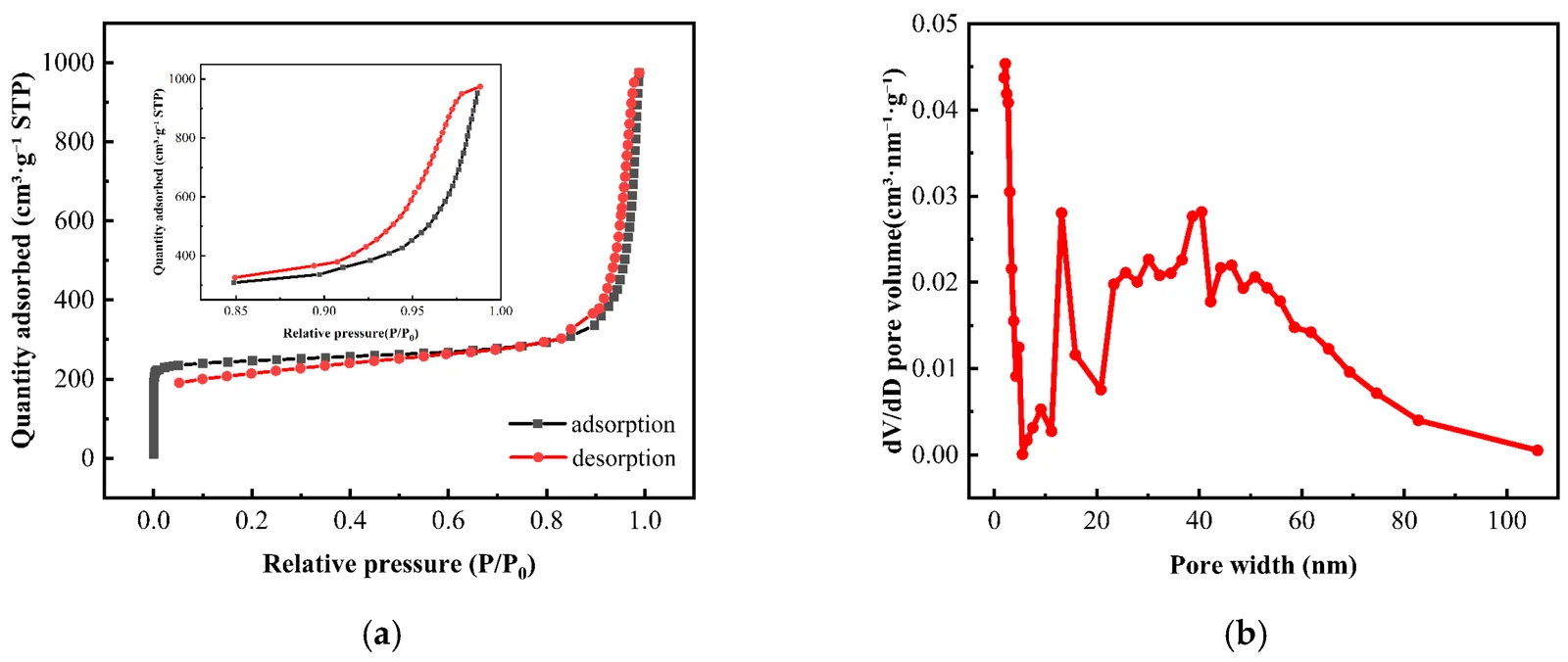
(**a**) N_2_ adsorption–desorption isotherms and (**b**) pore size distribution of NH_2_-MIL-53(Al).

N_2_ adsorption–desorption isotherms were carried out to analyze the pore structure properties of as-prepared NH_2_-MIL-53(Al), as displayed in Figure 2. The obtained isotherm exhibits the combined Type I and Type IV hybrid characteristics according to IUPAC classification. The steep adsorption uptake at a low relative pressure (P/P_0_ < 0.1) indicates typical micropore filling behavior, while a distinct hysteresis loop in the range of 0.7 < P/P_0_ < 1.0 confirms abundant mesopore formation, demonstrating the coexistence of intrinsic micropores and mesopores [27]. The reported average pore width of 19.51 nm is the BJH-weighted average value, dominated by the mesopore dimension. This statistical average parameter does not exclude the contribution of micropores, which well accounts for the coexisting micro–mesoporous architecture of the material. Specifically, inherent micropores originate from the intrinsic crystal framework of NH_2_-MIL-53(Al), while broadly distributed mesopores are derived from both intra-crystal defects and inter-particle stacking voids of aggregated nanocrystals. Macroporous gaps above 50 nm are mainly generated from nanocrystal stacking effects. Relevant pore-structure data are summarized in Table 1. The calculated pore parameters delivered a BET surface area of 794.18 m^2^·g^−1^, total pore volume of 1.51 cm^3^·g^−1^, and average channel width of 19.51 nm. For adsorptive materials, hierarchical pore features collectively offer accessible active sites and mass-transport pathways for target organic pollutants, jointly facilitating adsorption. The multilevel pore structure can reduce the mass transfer resistance for macromolecular contaminants to a certain extent, while uniformly distributed active amino groups provide abundant adsorption sites, laying a solid structural foundation for pollutant adsorption [28]. Nevertheless, direct visualization of the internal pore architecture by TEM/HRTEM is required in future investigations [29].

### 3.2. Adsorption Performance Under Variable Experimental Conditions

#### 3.2.1. Influence of Adsorbent Dosage and Initial Dye Concentration

**Figure 3:**
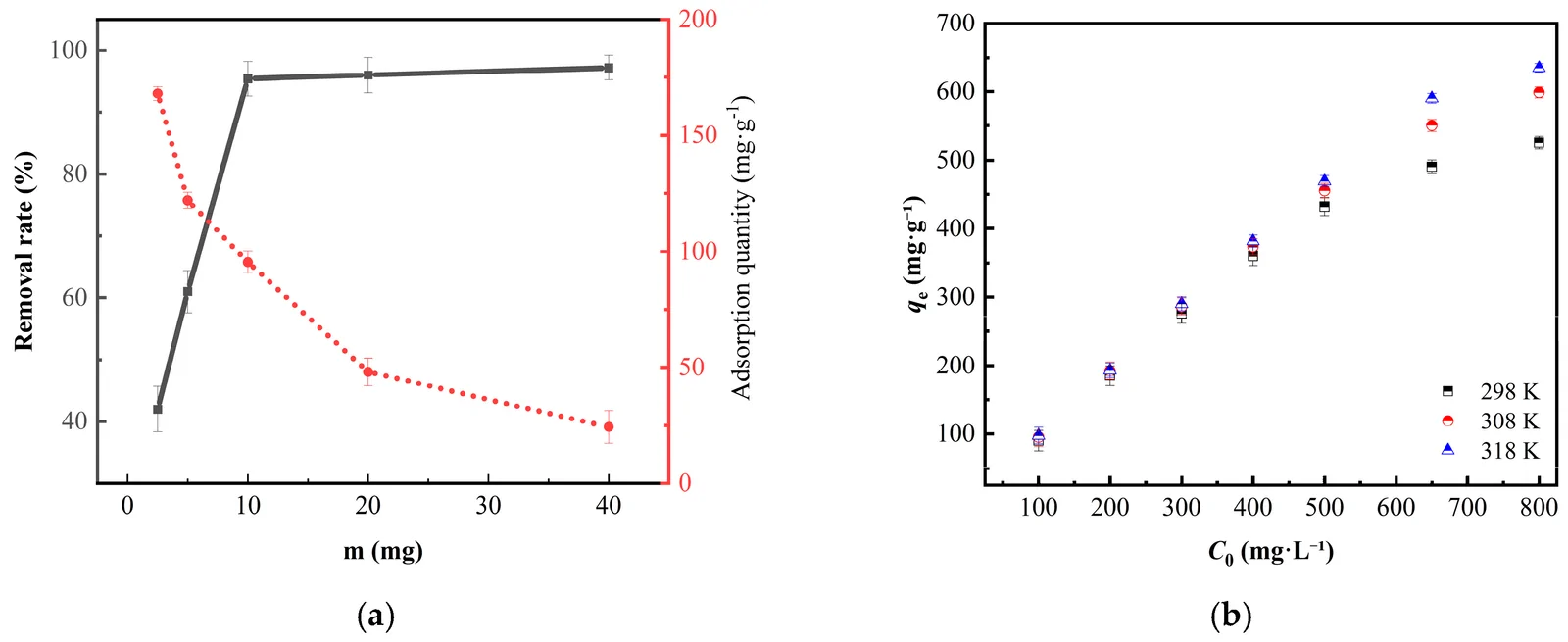
The effect of (**a**) adsorbent dosage and (**b**) initial dye concentration for the adsorption of Direct Scarlet dye onto NH_2_-MIL-53(Al).

The influence of adsorbent dosage on dye removal efficiency and unit adsorption capacity is illustrated in Figure 3a, under a fixed initial dye concentration of 300 mg·L^−1^ and a temperature of 308 K. When the dosage increased from 2.5 mg to 40 mg, the total available amino active sites multiplied, lifting removal efficiency from 42.0% to 97.2%. Nevertheless, excessive solid loading caused particle overlapping and partial shielding of nanochannels, leading to the unit capacity dropping from 168.0 mg·g^−1^ to 24.3 mg·g^−1^. A dosage of 10 mg was selected for subsequent tests to balance removal efficiency and material utilization.

The correlation between initial dye concentration and equilibrium capacity at three gradient temperatures is plotted in Figure 3b. Initial dye concentration determined the mass transfer driving force; adsorption capacity rose continuously and reached saturation above 650 mg·L^−1^ as all internal and surface active sites were occupied. In addition, adsorption capacity increases synchronously with reaction temperature, preliminarily proving the endothermic nature of this adsorption process, consistent with subsequent thermodynamic calculation results. At 318 K and an initial dye concentration of 800 mg·L^−1^, the actual equilibrium adsorption capacity of the material reaches 635.01 mg·g^−1^.

#### 3.2.2. Adsorption Kinetics

Adsorption kinetics, isotherms, and thermodynamics were systematically investigated to clarify the adsorption characteristics and dominant mechanism of Direct Scarlet dye onto hierarchically porous NH_2_-MIL-53(Al) [30]. Relevant mathematical fitting formulas are supplied in the Supporting Information, while all calculated parametric data from model fitting are compiled in Table 2, Table 3, Table 4 and Table 5.

**Figure 4:**
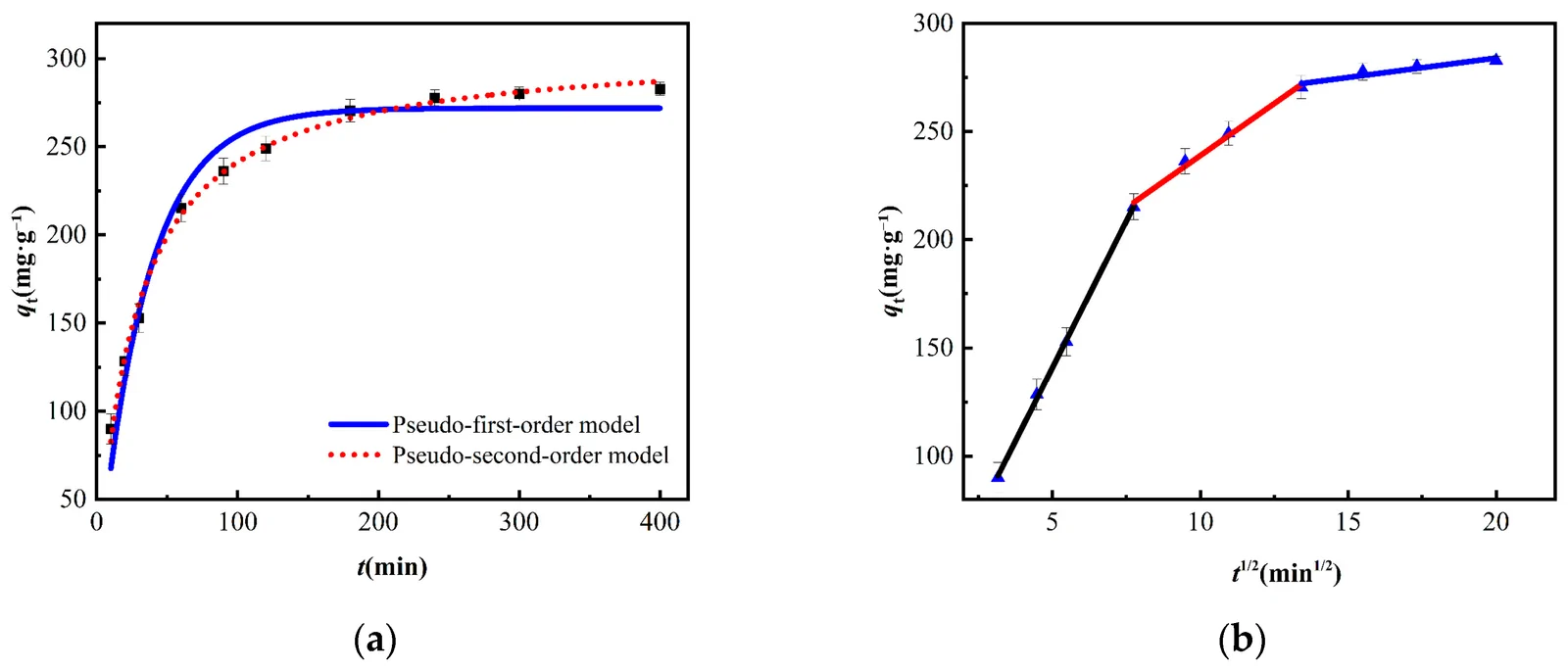
(**a**) Pseudo-first-order model and pseudo-second-order model; (**b**) Weber–Morris diffusion model for the adsorption of Direct Scarlet dye onto NH_2_-MIL-53(Al).

Time-variant adsorption behavior illustrated a typical uptake trend, as depicted in Figure 4a. Dye molecules were rapidly captured by the adsorbent within the first 120 min. Such fast adsorption behavior was closely related to the well-exposed amino functional sites and unobstructed hierarchical nanochannels of NH_2_-MIL-53(Al), which allowed sufficient contact between the adsorbent and dye solution. As active sites were gradually occupied, the adsorption rate slowed down continuously. The entire adsorption system finally achieved thermodynamic equilibrium at 240 min, yielding a maximum experimental adsorption capacity of 286.43 mg·g^−1^.

Three classical kinetic models, including pseudo-first-order, pseudo-second-order, and Weber–Morris intra-particle diffusion models, were employed to analyze the kinetic adsorption behavior (Figure 4a,b). The fitted kinetic parameters are summarized in Table 2 and Table 3. According to the fitting results, the pseudo-second-order model exhibited the highest correlation coefficient (*R*^2^ = 0.9953). Moreover, its theoretically calculated equilibrium adsorption capacity of 306.33 mg·g^−1^ showed great consistency with the experimentally measured value. This suggests that the adsorption behavior of Direct Scarlet dye onto hierarchically porous NH_2_-MIL-53(Al) is better predicted with a pseudo-second-order kinetic model, implying that the rate-controlling step in this adsorption process might be chemisorption [10,31,32,33,34].

The Weber–Morris intra-particle diffusion model was further adopted to identify the detailed mass transfer steps and rate-limiting factors. The fitting curves were divided into three independent linear stages and failed to pass through the coordinate origin, confirming that intra-particle diffusion was not the sole factor controlling the adsorption rate [35]. The first linear stage corresponded to fast external surface adsorption, where dye molecules quickly diffused from the bulk solution to the material surface. The second stage, with a slower diffusion rate, was attributed to intra-particle pore diffusion, which acted as the major rate-limiting step throughout the adsorption process due to gradually increased mass transfer resistance. The final platform stage represented the dynamic equilibrium state, in which the adsorption and desorption rates reached a relative balance. Owing to its interconnected micro–mesoporous structure, NH_2_-MIL-53(Al) effectively shortened the molecular diffusion distance and facilitated mass transfer efficiency. Overall, the synergistic effect of accessible amino active sites and hierarchical porous structure endows the material with excellent dye adsorption performance.

#### 3.2.3. Equilibrium Adsorption Isotherms

**Figure 5:**
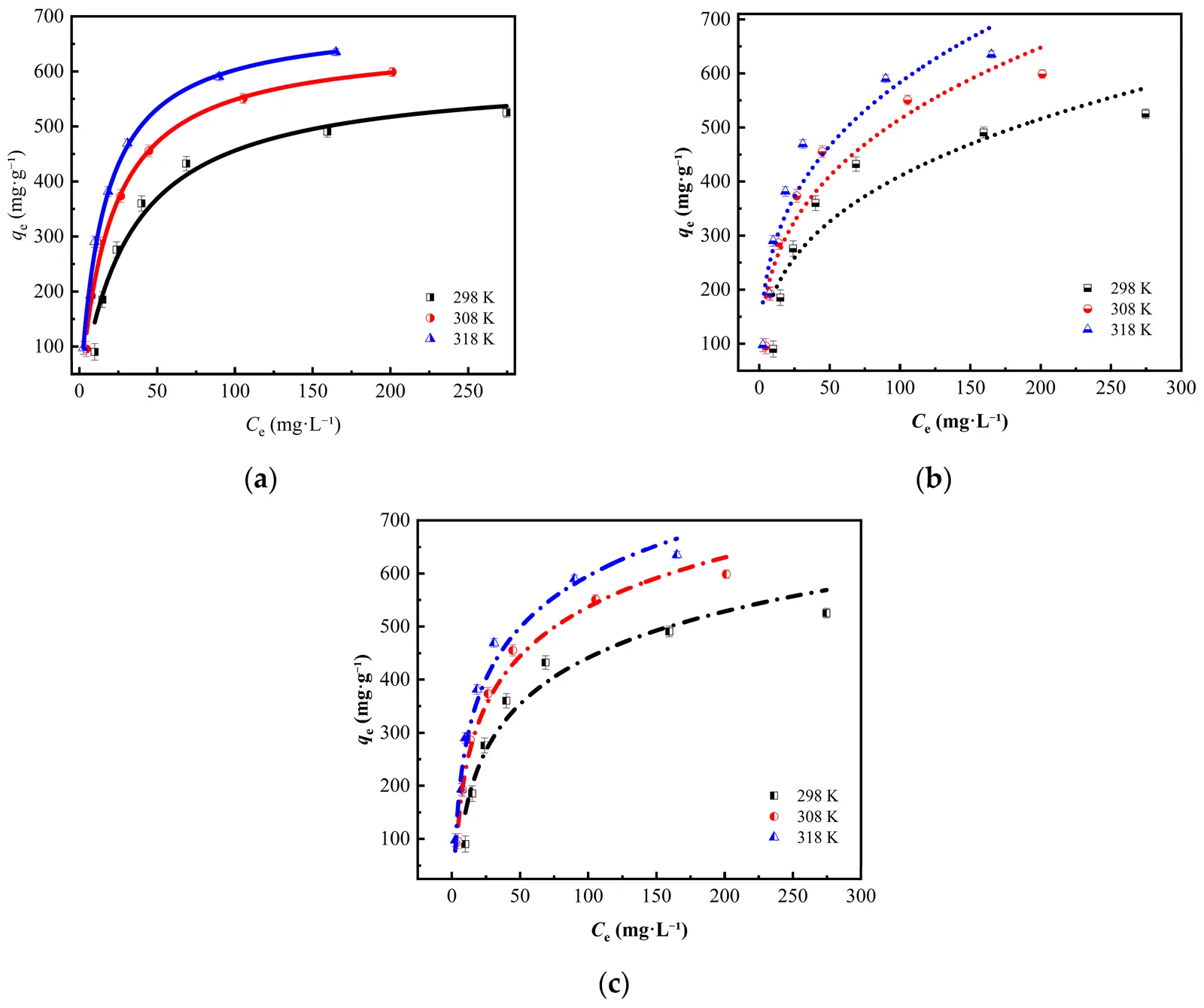
(**a**) Langmuir, (**b**) Freundlich, and (**c**) Temkin isotherms for the adsorption of Direct Scarlet onto NH_2_-MIL-53(Al).

Adsorption isotherms were measured at 298 K, 308 K, and 318 K to elucidate the equilibrium adsorption behavior of Direct Scarlet dye on hierarchically porous NH_2_-MIL-53(Al). As illustrated in Figure 5, the equilibrium adsorption capacity increases with elevated dye concentration and gradually reaches saturation at high concentrations. Notably, the adsorption capacity increases continuously with rising temperature under identical equilibrium concentrations, verifying the endothermic nature of the adsorption reaction.

Three classical isotherm models, including Langmuir, Freundlich, and Temkin models, were adopted to fit the equilibrium data, with corresponding fitted curves displayed in Figure 5 and relevant parametric results summarized in Table 4. Across the whole temperature range, the Langmuir model presents comparatively higher correlation coefficients (*R*^2^ = 0.9660–0.9943), as well as lower SSE (sum of squared errors) and RMSE (root mean square error) values compared with the Freundlich and Temkin models. The combination of higher correlation and smaller residual errors confirms that the Langmuir model provides the optimal mathematical fitting for the measured equilibrium data, indicating that the adsorption process follows a typical monolayer adsorption on the homogeneous surface of NH_2_-MIL-53(Al) [21]. Moreover, the *R*^2^ values of the Langmuir model increase steadily with rising temperature, suggesting that elevated temperature favors the adsorption behavior of the dye on NH_2_-MIL-53(Al).

The maximum adsorption capacity calculated from the Langmuir model reaches 694.99 mg·g^−1^ at 318 K, which is superior to that of defective Zr-based MOFs reported in our previous research for Direct Scarlet dye removal.

Although the Freundlich constant 1/n ranges from 0.3242 to 0.3314 within the favorable adsorption interval of 0.1–0.5, suggesting the spontaneous and facile nature of the dye adsorption process [35], the relatively low fitting result of the Freundlich model reveals that multilayer adsorption is not the dominant adsorption pathway in this adsorption process. In addition, the fitting results of the Temkin model indicate negligible heat variation during the whole adsorption procedure, which further verifies that weak electrostatic interaction serves as the primary intermolecular force between NH_2_-MIL-53(Al) and Direct Scarlet dye molecules.

#### 3.2.4. Thermodynamic Analysis

**Figure 6:**
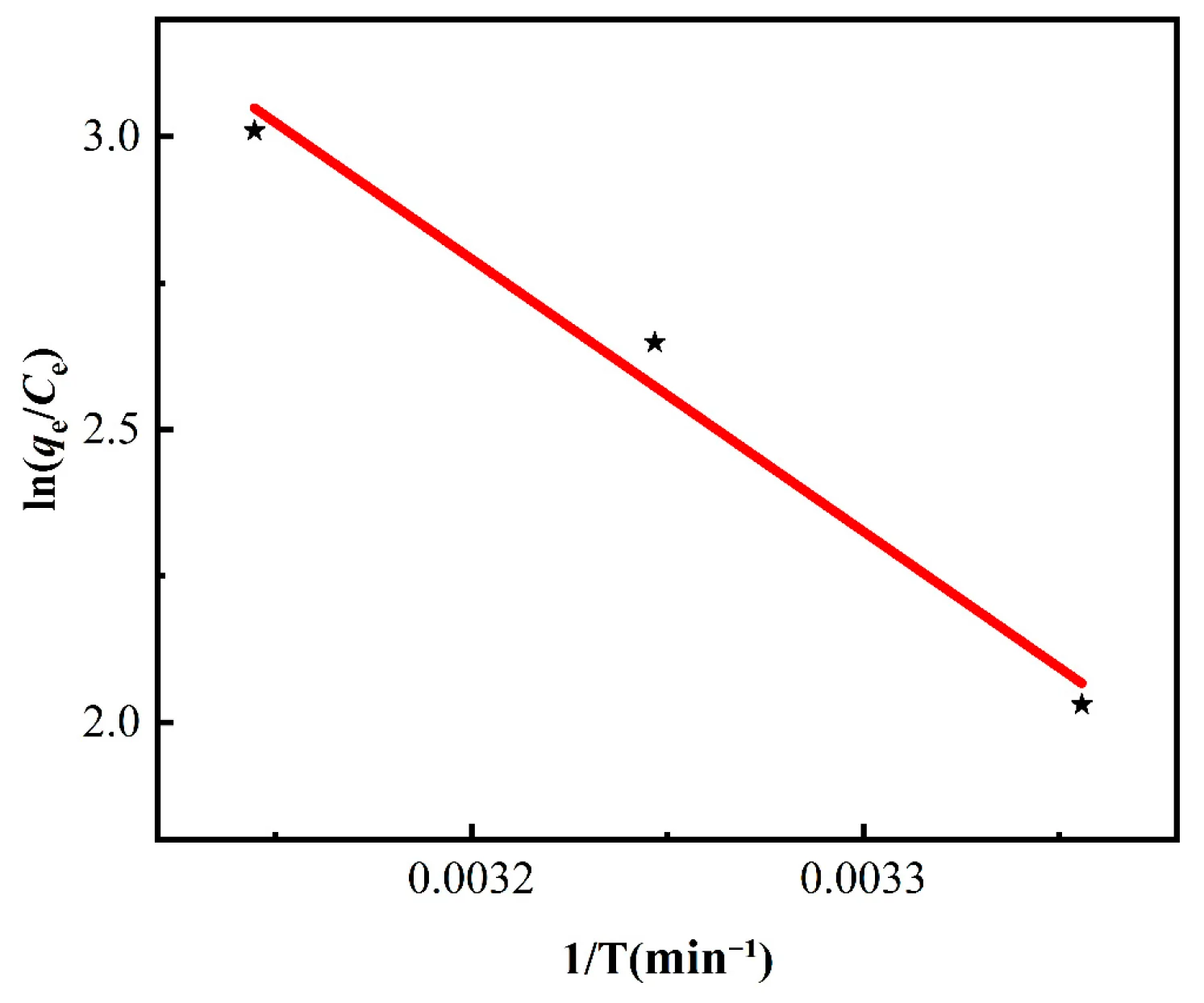
Thermodynamic analysis for adsorption of Direct Scarlet dye onto NH_2_-MIL-53(Al).

The thermodynamic characteristics of Direct Scarlet dye adsorption on hierarchically porous NH_2_-MIL-53(Al) were investigated at three different temperatures, and the corresponding fitting plot for thermodynamic parameter calculation is presented in Figure 6. All key thermodynamic parameters, including enthalpy change (ΔH), entropy change (ΔS), and Gibbs free energy change (ΔG), are statistically summarized in Table 5.

The positive ΔH value of 38.66 kJ·mol^−1^ clearly demonstrates the endothermic nature of the adsorption process, which is highly consistent with the elevated adsorption capacity observed at higher temperatures in isotherm experiments in Figure 5 [21,31]. This endothermic behavior originates from the energy consumption during the dehydration of dye molecules and the desolvation of interfacial ions, as well as the formation of stable intermolecular interactions between the adsorbent and dye molecules.

The positive ΔS value verifies the increased randomness and disorder of the solid–liquid interface after dye molecules diffuse into the hierarchical nanochannels of NH_2_-MIL-53(Al), indicating the favorable interfacial mass transfer during adsorption. All ΔG values calculated within the tested temperature range are negative, confirming the spontaneous and thermodynamically favorable feature of the dye adsorption process [36]. Moreover, the absolute value of ΔG increases gradually with rising temperature, further proving that high temperature can effectively promote the capture of Direct Scarlet dye [37].

Combined with the FTIR spectral analysis and previous model fitting results, the ΔG range of −5 to −8 kJ·mol^−1^ further corroborates that electrostatic chemisorption acts as the dominant interaction mechanism throughout the adsorption process, which maintains consistent conclusions with kinetic and isotherm analysis.

#### 3.2.5. Recyclability of Hierarchical NH_2_-MIL-53(Al)

**Figure 7:**
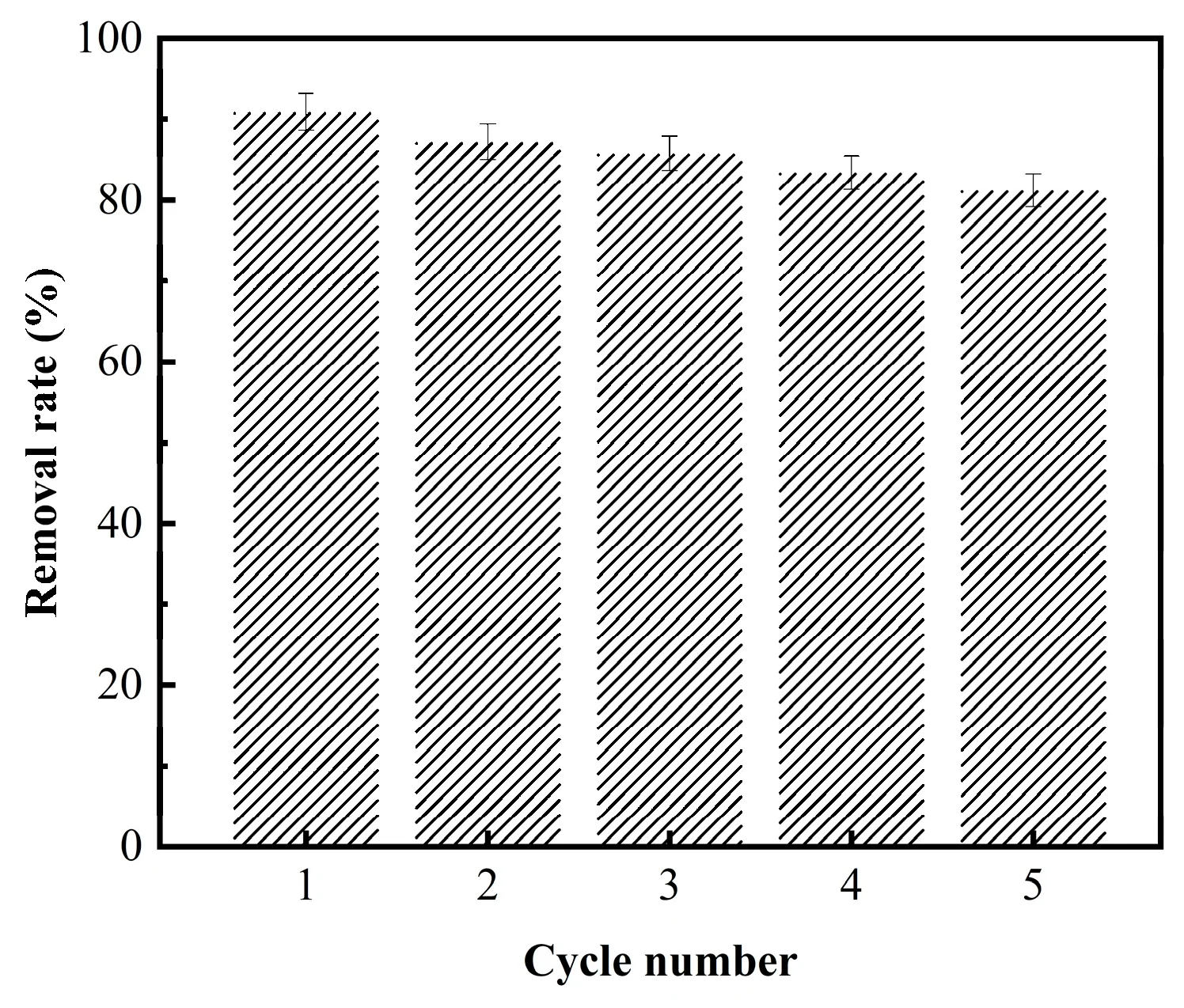
Reusability of NH_2_-MIL-53(Al) for adsorption of Direct Scarlet dye.

Five consecutive adsorption–desorption cyclic experiments were implemented to systematically evaluate the reusability and performance of hierarchically porous NH_2_-MIL-53(Al) for Direct Scarlet dye removal, and the corresponding cyclic performance results are summarized in Figure 7. After five complete regeneration cycles, the synthesized nanosorbent still maintained more than 85% of its initial adsorption capacity, demonstrating satisfactory cyclic usability.

**Figure 8:**
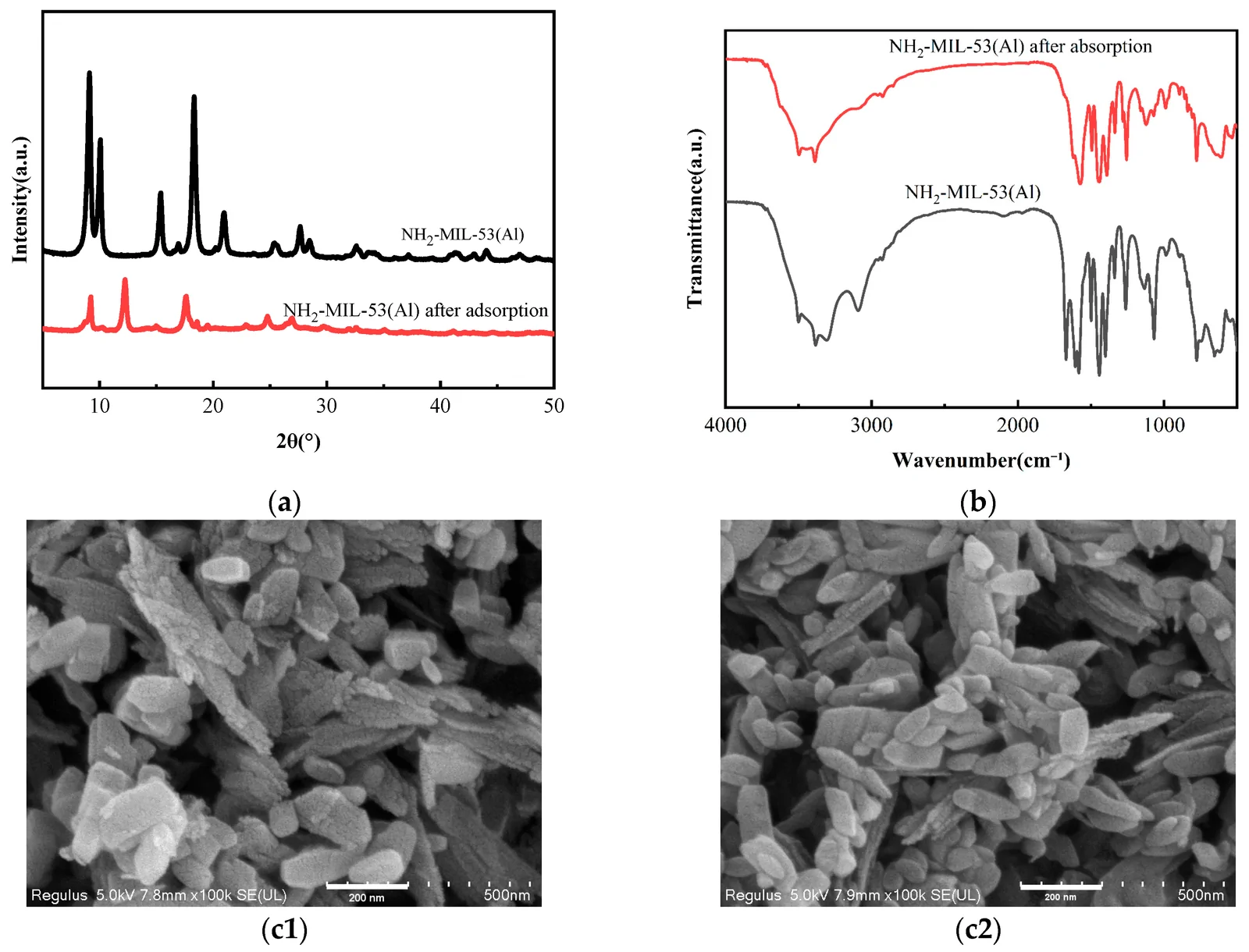
(**a**) XRD patterns, (**b**) FT-IR spectra, and (**c1**,**c2**) SEM of NH_2_-MIL-53(Al) before and after Direct Scarlet dye adsorption ((**c1**) NH_2_-MIL-53(Al) before Direct Scarlet dye adsorption, (**c2**) NH_2_-MIL-53(Al) after Direct Scarlet dye adsorption).

To further verify the structural stability of the recycled material, multiple microscopic characterizations including PXRD, FTIR, and FE-SEM were conducted, and the relevant results are displayed in Figure 8a–c. The cycled NH_2_-MIL-53(Al) samples retained complete characteristic diffraction peaks with no obvious peak shift or intensity attenuation, confirming the well-preserved crystalline framework. Meanwhile, the intrinsic rod-like nano-morphology of the material remained intact without particle pulverization or severe inter-particle aggregation after repeated adsorption and elution processes. These structural stabilities demonstrate that the 70% ethanol aqueous solution can effectively strip dye molecules trapped in the interconnected hierarchical nanochannels, while maintaining the integrity of the Al–O coordination skeleton of NH_2_-MIL-53(Al) [23].

Compared with the defect-engineered NH_2_-UiO-66 reported in our previous work, which suffers from irreversible lattice damage and continuous performance attenuation after multiple cycles due to artificial ligand defects, NH_2_-MIL-53(Al) possesses a stable native flexible tunnel topology. Such unique structural characteristics endow the material with excellent cyclic stability, making it a promising candidate for further investigation toward textile wastewater treatment.

### 3.3. Adsorption Mechanism Analysis

The adsorption mechanism of Direct Scarlet dye on hierarchically porous NH_2_-MIL-53(Al) was clarified by correlating structural evolution, interfacial molecular interactions, and thermodynamic and kinetic behaviors, based on comparative PXRD, FTIR, and FE-SEM characterizations of pristine and post-adsorption samples, as presented in Figure 8.

The PXRD patterns presented negligible differences before and after dye adsorption, indicating that the infinite Al–O inorganic chain framework of NH_2_-MIL-53(Al) was well preserved after the uptake of large azo dye molecules [36]. The flexible topological tunnels of MIL-53 underwent only reversible structural expansion to accommodate dye guests, rather than irreversible lattice destruction. Such reversible framework breathing endows the material with a stable structural foundation for cyclic adsorption and desorption, which accounts for its satisfactory recyclability during repeated wastewater treatment.

FTIR spectra further verified the interfacial interaction between the NH_2_-MIL-53(Al) adsorbent and dye molecules. After adsorption, the characteristic stretching vibration of amino groups at approximately 3390 cm^−1^ was significantly weakened, while new absorption bands belonging to the S=O stretching vibration of sulfonate groups emerged within 1000–1200 cm^−1^. Under neutral aqueous conditions, surface amino groups were readily protonated to produce positively charged –NH_3_^+^ active sites, which can strongly anchor the negatively charged –SO_3_^−^ groups distributed on Direct Scarlet dye molecules via electrostatic attraction [33]. This spectroscopic signature, together with the negative Gibbs free-energy values ΔG ranging from −5 to −8 kJ·mol^−1^ obtained from the thermodynamic analysis and the monolayer adsorption behavior described by the Langmuir isotherm model, jointly supports electrostatic-dominated chemisorption as the major interaction contributing to dye uptake. Nevertheless, an ideal homogeneous adsorption surface cannot be fully achieved, owing to the inherent structural defects and hierarchical micro–mesoporous architecture of the synthesized NH_2_-MIL-53(Al) adsorbent. Other interactions such as hydrogen-bonding may also participate in the overall dye-capture process.

FE-SEM observations in Figure 8c demonstrated that the original rod-like aggregated microstructure of NH_2_-MIL-53(Al) remained intact after dye saturation. No structural collapse, surface obstruction, or severe pore blockage was observed, indicating that the interconnected micro–mesoporous network was well maintained. Such unobstructed multilevel channels guarantee efficient and stable mass transfer during continuous dye adsorption and solvent elution.

Combined with the structural pore parameters (Figure 2, Table 1) and isotherm fitting results (Table 4), the unique hierarchical nanochannels derived from the intrinsic breathing effect of MIL-53 effectively reduce intra-particle diffusion resistance and expose abundant internal amino active sites. This structural advantage fundamentally accounts for the rapid adsorption kinetics and high saturated capacity of NH_2_-MIL-53(Al). Consistent with Langmuir monolayer adsorption characteristics, electrostatic binding sites are uniformly distributed on the inner tunnel surface, enabling homogeneous and stable dye immobilization. Benefiting from the reversible expansion–contraction behavior of the Al–O framework, dye molecules can be readily captured and eluted without permanent structural damage, further explaining the excellent cyclic stability of the material. The synergistic mechanism between porous structure and amino functionalization fully illustrates the superior dye removal performance of the prepared NH_2_-MIL-53(Al) adsorbent.

### 3.4. Comparative Adsorption Performance with Previously Reported Adsorbents

To objectively evaluate the comprehensive application advantages of hierarchical NH_2_-MIL-53(Al), its Langmuir maximum adsorption capacity for Direct Scarlet dye was compared with a variety of previously reported adsorbents, and the detailed comparative data are listed in Table 6. All literature adsorption values summarized in Table 6 were obtained under their respective experimental conditions. The maximum adsorption capacity of NH_2_-MIL-53(Al) reaches 694.99 mg·g^−1^ at 318 K, which is comparable to that of most reported biomass materials, porous organic polymers, and single-microporous MOFs, whose adsorption capacities are generally lower than 400 mg·g^−1^.

Different from defective NH_2_-UiO-66 in our previous research, which only possesses isolated and discrete mesoporous cavities, NH_2_-MIL-53(Al) features intrinsically interconnected micro–mesoporous nanochannels. Such continuous mass-transport pathways are constructed on intact crystalline lattices, effectively eliminating structural defects and maximizing the accessibility of internal active sites. Homogeneously distributed amino groups can be sufficiently protonated under neutral aqueous conditions, generating abundant positive adsorption centers for the efficient capture of anionic Direct Scarlet dye molecules.

Beyond its prominent adsorption capacity, NH_2_-MIL-53(Al) exhibits remarkable practical usability in terms of synthetic feasibility and cyclic durability. The material can be easily fabricated via a one-pot solvothermal route and maintains more than 85% of its original dye removal efficiency after five regeneration cycles. In contrast, defect-modified MOFs suffer from irreversible lattice deterioration and rapid performance decay during repeated cycling, severely limiting their practical applicability. This defect-free structural strategy provides a reliable crystal engineering avenue for designing high-performance nanosorbents, showing encouraging potential for dye-contaminated wastewater remediation.

## 4. Conclusions

This work reports hierarchically porous NH_2_-MIL-53(Al) nanocrystals as high-efficiency adsorbents for anionic Direct Scarlet dye removal. The synthesized material exhibits a favorable micro–mesoporous structure and excellent structural stability. Combined isotherm, thermodynamic, kinetic, and spectroscopic analyses reveal spontaneous, endothermic, predominant monolayer adsorption behavior, while partial surface heterogeneity originating from structural defects cannot be excluded. The material delivers a prominent Langmuir maximum adsorption capacity of 694.99 mg·g^−1^ at 318 K toward Direct Scarlet. After five laboratory-scale adsorption–desorption cycles, the material retains over 85% dye removal efficiency, and PXRD, FTIR, and FE-SEM characterizations confirm the well-preserved crystalline framework and nanorod morphology. This study highlights the advantages of the pore-engineering strategy for fabricating high-performance MOF-based adsorbents and presents promising potential toward textile dye wastewater treatment, while further evaluation with more regeneration cycles and real wastewater samples is required for practical deployment.

## Figures and Tables

**Table 1:** Porosity properties of NH_2_-MIL-53(Al).

Sample	S_BET_ (m^2^·g^−1^)	V_micro_ (cm^3^·g^−1^)	V_total_ (cm^3^·g^−1^)	Pore Size (nm)
NH_2_-MIL-53(Al)	794.18	0.32	1.51	19.51

**Table 2:** Adsorption kinetics parameters for NH_2_-MIL-53(Al).

Adsorbent	Pseudo-First-Order Model			Pseudo-Second-Order Model		
	*q*_e_ (mg·g^−1^)	*k*_1_ (min^−1^)	*R* ^2^	*q*_e_ (mg·g^−1^)	*k*_2_ (g·mg^−1^·min^−1^)	*R* ^2^
NH_2_-MIL-53(Al)	271.87	0.02856	0.9664	306.33	0.001210	0.9953

**Table 3:** The Weber–Morris diffusion kinetic model fitting parameters.

*C*_0_ (mg·L^−1^)		Weber–Morris Diffusion Kinetic Model				
	*k*_1_ (mg·g^−1^·min^−1/2^)	*R* ^2^	*k*_2_ (mg·g^−1^·min^−1/2^)	*R* ^2^	*k*_3_ (mg·g^−1^·min^−1/2^)	*R* ^2^
300	27.1242	0.9989	9.6232	0.9891	1.7944	0.8486

**Table 4:** Isotherm fitting parameters.

T (K)	Langmuir Model				Freundlich Model					Temkin Model				
	*q*_m_ (mg·g^−1^)	*K*_L_ (L·mg^−1^)	*R* ^2^	SSE	RMSE	*K*_F_ (L·mg^−1^)	1/n	*R* ^2^	SSE	RMSE	*b*_T_ (J·mol^−1^)	*R* ^2^	SSE	RMSE
298	597.74	0.03216	0.9660	4427.24	29.76	89.14	0.3314	0.8299	22,169.79	66.59	19.30	0.9364	10,068.53	37.93
308	656.47	0.05081	0.9928	1236.25	15.72	112.82	0.3298	0.8946	18,101.11	60.19	19.09	0.9820	3088.69	24.85
318	694.99	0.06506	0.9943	1136.51	15.08	131.06	0.3242	0.9017	19,667.31	62.72	19.49	0.9789	6304.94	30.01

**Table 5:** Thermodynamic parameters for the adsorption of Direct Scarlet dye onto NH_2_-MIL-53(Al).

Δ*H*	Δ*S*	Δ*G* (kJ·mol^−1^)			*R* ^2^
(kJ·mol^−1^)	(kJ·mol^−1^)	298 K	308 K	318 K	
38.6662	0.1469	−5.1209	−6.5903	−8.0597	0.9659

**Table 6:** Maximum adsorption capacity comparison with reported adsorbents.

Adsorbent	*q*_m_ (mg·g^−1^)	Experiment Condition	References
Hydrazide-linked perylene-based POP	87.50	pH = 4, c0 = 250 ppm, adsorbent dosage = 5 mg, V = 5 mL, 25 °C, stirred for 4 h	[38]
MNP@m-CTP	215.28	pH = 3, c0= mg·L^−1^, adsorbent dosage = 3 mg, V = 4 mL, room temperature, stirred for 4 h	[39]
MDA-POP	136.97	pH = 4, c0 = 300 mg·L^−1^, adsorbent dosage = 6 mg, V = 5 mL, room temperature, stirred for 1 h	[40]
FC-POP-EDA@Fe_3_O_4_	32.13	pH = 2, c0 = 200 mg·L^−1^, adsorbent dosage = 40 mg, V = 5 mL, room temperature, shaken for 4 h	[41]
Acid-treated *E. prolifera* (ATEP)	318.87	pH, 2; ATEP dosage = 4 g·L^−1^; 333 K, shaking time, 7 h	[42]
TA-TG_Cl_	236.40	pH = 2, c0 = 250 ppm, adsorbent dosage = 5 mg, V = 5 mL, room temperature, time = 4 h	[43]
Defective NH_2_-UiO-66	534.76	pH = 7, c0 = 800 mg·L^−1^, adsorbent dosage = 10 mg, V = 10 mL, 318 K, time = 4 h	[21]
Hierarchically porous NH_2_-MIL-53(Al)	694.99	pH = 7, c0 = 650 mg·L^−1^, adsorbent dosage = 10 mg, V = 10 mL, 318 K, time = 4 h	This work

## Data Availability

The original contributions presented in this study are included in the article/Supplementary Material. Further inquiries can be directed to the corresponding author.

## Supplementary Materials

The following supporting information can be downloaded at: https://www.mdpi.com/article/10.3390/nano16181135/s1, Figure S1: Adsorption capacities of MIL-53(Al) and NH_2_-MIL-53(Al) for Direct Scarlet dye; Mathematical Formulas (S1)–(S11); Table S1: SSE and RMSE of adsorption isotherms.
