# Supplementary material for: NH2-MIL-53(Al) with Hierarchical Micro–Mesoporosity for Highly Efficient Capture of Direct Scarlet Anionic Dye: Insights into Structure–Property Correlations
Source: Nanomaterials (Basel). 2026 Sep 10;16(18):1135. doi: 10.3390/nano16181135 (PMC13609969; doi:10.3390/nano16181135)
Supplement: Supplementary file 1 [file nanomaterials-16-01135-s001.zip › nanomaterials-4532566-supplementary.pdf]

nanomaterials

MDPI

Supporting Information

NH_2-MIL-53 (Al) with Hierarchical Micro–Mesoporosity for Highly Efficient Capture of Direct Scarlet Anionic Dye: Insights into Structure–Property Correlations

Ruiming Zhao [1] and Lingling Li [2,*]

1 Center of Analysis and Testing, Guangdong University of Petrochemical Technology, Maoming 525000, China; rmzhao@gdupt.edu.cn
2 School of Chemical Engineering, Guangdong University of Petrochemical Technology, Maoming 525000, China
* Correspondence: linglingli@gdupt.edu.cn

S1. Pore Structure Calculation Methods

N_2 adsorption–desorption isotherms were measured to evaluate the porous structure of NH_2-MIL-53(Al). The micropore volume and micropore specific surface area were calculated via the t-plot method, and the micropore size distribution was determined using the Horvath–Kawazoe (HK) model. The mesopore size distribution was obtained by the Barrett–Joyner–Halenda (BJH) algorithm based on the desorption branch of the isotherm. The total pore volume (Vtotal) was determined at a relative pressure of $P/P_0 \approx 0.99$. The mesopore volume (Vmeso) was calculated by subtracting the micropore volume (Vmicro) from the total pore volume (Vmeso = Vtotal − Vmicro). The average pore width was defined as the BJH-weighted average pore size of the micro–mesopore system.

S2. Mathematical equation:

The dye adsorption capacity and removal efficiency were calculated using the following equations:

$$q_e = \frac{(C_0 - Ce) * V}{m} \quad \text{(S1)}$$

$$R\% = \frac{(C_0 - Ce)}{C_0} * 100\% \quad \text{(S2)}$$

Where q_e ($mg \cdot g^{-1}$) is the equilibrium adsorption capacity of Direct Scarlet dye, m (g) is the mass of the adsorbent, V(L) is the volume of the Direct Scarlet dye aqueous solution, C_0($mg \cdot L^{-1}$) is the initial concentration of Direct Scarlet dye, C_e ($mg \cdot L^{-1}$) is the concentration at equilibrium, and R (%) is the removal efficiency of Direct Scarlet dye.

S3. Adsorption Kinetic

Kinetic studies are essential for understanding the adsorption mechanism and determining key parameters such as rate constants. Experimental data for Direct Scarlet dye adsorption on NH_2-MIL-53(Al) were fitted with the common kinetic models:

Pseudo-first-order model:

$$q_t = q_e(1 - e^{-k_1 t}) \quad \text{(S3)}$$

Pseudo-second-order model:

$$q_t = \frac{k_2 q_e^2 t}{1 + k_2 q_e t} \quad \text{(S4)}$$

Additionally, the Weber–Morris intra-particle diffusion model was considered:

$$q_t = k_{pi} t^{1/2} + C \quad \text{(S5)}$$

Where q_e and q_t are the adsorption capacity (mg·g^{-1}) at equilibrium and time t (min), respectively; k_1 and k_2 are the rate constants for the pseudo-first-order (min^{-1}) and pseudo-second-order models (g·mg^{-1}·h^{-1}). k_{pi} (g·mg^{-1}·min$^{-1/2}$) represents the intra-particle diffusion rate constant, and C is a constant related to diffusion resistance.

S4. Adsorption Isotherm

Equilibrium adsorption data were analyzed using Langmuir, Freundlich, and Temkin isotherm models. The corresponding non-linear equations are presented below.

Langmuir model:

$$q_\mathrm{e} = \frac{q_\mathrm{m} K_\mathrm{L} C_\mathrm{e}}{1+\mathrm{K}_\mathrm{L} C_\mathrm{e}} \quad \text{(S6)}$$

Freundlich model:

$$q_\mathrm{e} = K_\mathrm{F} C_\mathrm{e}^{\frac{1}{\mathrm{n}}} \quad \text{(S7)}$$

Temkin model:

$$q_\mathrm{e} = \frac{RT}{b_\mathrm{T}} \ln A_\mathrm{T} + \frac{RT}{b_\mathrm{T}} \ln c_\mathrm{e} \quad \text{(S8)}$$

Where q_e (mg·g^{-1}) and C_e (mg·L^{-1}) are the equilibrium adsorption capacity and the equilibrium concentration of Direct Scarlet dye. For the Langmuir isotherm, q_m (mg·g^{-1}) is the theoretical saturated adsorption capacity of the monolayer and K_L (L·mg^{-1}) is the Langmuir adsorption constant related to the adsorption energy of the binding site. For the Freundlich isotherm, K_F (mg·g^{-1}) and n are Freundlich constants representing adsorption capacity and intensity, respectively. In the Temkin model, b_T (J·mol^{-1}) is the Temkin constant related to heat of adsorption, A_T (L·mg^{-1}) is the equilibrium binding constant, R is the universal gas constant (8.314 J·mol^{-1}·K^{-1}), and T (K) is the absolute temperature.

S5. Adsorption Thermodynamics

To gain insights into the thermodynamic behavior of the adsorption process, experiments were conducted over a range of temperatures (298 K, 308 K, and 318 K). Key parameters such as changes in enthalpy (ΔH), entropy (ΔS), and Gibbs energy (ΔG) were calculated using the following equations:

$$\triangle G = \triangle H - \mathrm{T} \triangle S \quad \text{(S9)}$$

$$\ln K_d = -\frac{\triangle H}{\mathrm{RT}} + \frac{\triangle S}{\mathrm{R}} \quad \text{(S10)}$$

$$K_\mathrm{d} = \frac{q_\mathrm{e}}{C_\mathrm{e}} \quad \text{(S11)}$$

Where R is the gas constant, equal to 8.314 J·mol^{-1}·K^{-1}; T is the temperature in Kelvin (K). K_d is the distribution coefficient, C_e (mg·L^{-1}) is the equilibrium concentration, and q_e(mg·g^{-1}) is the adsorption capacity.

S6. Adsorption Capacities

The adsorption capacities of MIL-53(Al) and hierarchically porous NH_2-MIL-53(Al) for Direct Scarlet dye were compared at 308 K, with results shown in Figure S1. For both materials, adsorption capacity increased with initial concentration. However, MIL-53(Al) plateaued at 400 mg·L^{-1}, with a maximum capacity of only 220 mg·g^{-1}. In contrast, NH_2-MIL-53(Al) exhibited a much higher adsorption capacity of 635.01 mg·g^{-1} at 800 mg·L^{-1}, which is attributed to the synergistic effect of amino functionalization and hierarchical porosity. The intrinsic microporous structure of MIL-53(Al) restricts the diffusion of bulky dye molecules and lacks sufficient active sites, resulting in low adsorption capacity. In comparison, the hierarchical porous structure of NH_2-MIL-53(Al) facilitates rapid mass transfer and provides abundant accessible active sites, while the amino groups form strong electrostatic interactions with anionic dye molecules, thus significantly enhancing the adsorption performance.

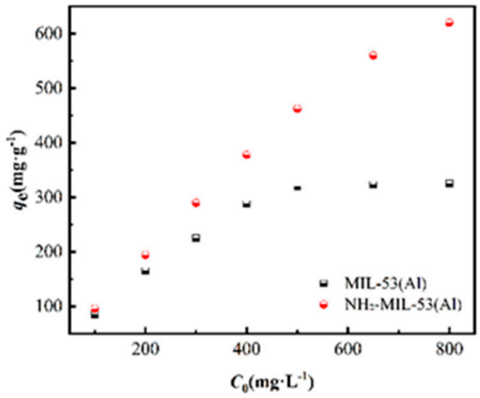

Figure S1. Adsorption capacities of MIL-53(Al) and NH_2-MIL-53(Al) for Direct Scarlet dye.

Table S1. SSE and RMSE of adsorption isotherms.

T	Langmuir model		Freundlich model		Temkin model	
(K)	SSE	RMSE	SSE	RMSE	SSE	RMSE
298	4427.24	29.76	22169.79	66.59	10068.53	37.93
308	1236.25	15.72	18101.11	60.19	3088.69	24.85
318	1136.51	15.08	19667.31	62.72	6304.94	30.01
